# High Reward Makes Items Easier to Remember, but Harder to Bind to a New Temporal Context

**DOI:** 10.3389/fnint.2012.00061

**Published:** 2012-08-27

**Authors:** Christopher R. Madan, Esther Fujiwara, Bridgette C. Gerson, Jeremy B. Caplan

**Affiliations:** ^1^Department of Psychology, University of AlbertaEdmonton, AB, Canada; ^2^Department of Psychiatry, University of AlbertaEdmonton, AB, Canada; ^3^Centre for Neuroscience, University of AlbertaEdmonton, AB, Canada

**Keywords:** reward, value, context, free recall, lexical decision, implicit memory, explicit memory

## Abstract

Learning through reward is central to adaptive behavior. Indeed, items are remembered better if they are experienced while participants expect a reward, and people can deliberately prioritize memory for high- over low-valued items. Do memory advantages for high-valued items only emerge after deliberate prioritization in encoding? Or, do reward-based memory enhancements also apply to unrewarded memory tests and to implicit memory? First, we tested for a high-value memory advantage in unrewarded implicit- and explicit-tests (Experiment 1). Participants first learned high or low-reward values of 36 words, followed by unrewarded lexical decision and free-recall tests. High-value words were judged faster in lexical decision, and more often recalled in free recall. These two memory advantages for high-value words were negatively correlated suggesting at least two mechanisms by which reward value can influence later item-memorability. The ease with which the values were originally acquired explained the negative correlation: people who learned values earlier showed reward effects in implicit memory whereas people who learned values later showed reward effects in explicit memory. We then asked whether a high-value advantage would persist if trained items were linked to a new context (Experiments 2a and 2b). Following the same value training as in Experiment 1, participants learned lists composed of previously trained words mixed with new words, each followed by free recall. Thus, participants had to retrieve words only from the most recent list, irrespective of their values. High- and low-value words were recalled equally, but low-value words were recalled earlier than high-value words and high-value words were more often intruded (proactive interference). Thus, the high-value advantage holds for implicit- and explicit-memory, but comes with a side effect: High-value items are more difficult to relearn in a new context. Similar to emotional arousal, reward value can both enhance and impair memory.

## 1 Introduction

When faced with items of differing reward values, an individual has the possibility of prioritizing their efforts to learn as much as possible about the higher-valued items, likely at the expense of knowledge about the lower-value items. If people took advantage of this, they could maximize their accumulation of reward. In seeking reward, it may not only be beneficial to remember the values of items, but also related information such as the precise context in which the item was found, which we refer to as the reward-maximization hypothesis. Alternatively, reward value may be emotionally arousing; thus, effects of reward value on memory may resemble those found with emotional arousal. Emotionally arousing items are generally remembered better, but memory for related contextual information is often impaired (Easterbrook, 1959; Burke et al., 1992; Christianson, 1992; Mather and Sutherland, 2011; Madan et al., 2012). Such impairment may be caused by diverting attention toward the arousing stimulus itself, and away from its context. If reward value functions like emotional arousal, then higher reward value should result in enhanced performance on some tests of memory (e.g., memory for the experienced items alone), but not others (e.g., judging whether an item was presented in a specific context), which we refer to here as the value-interference hypothesis. Whether higher reward value universally results in better item-memory across different types of memory tests (explicit and implicit), and whether reward value results in better memory for context is unknown. Finding a benefit for high-value items in rewarded memory tests tells us that participants are capable of prioritizing high-value items, but leaves open the question of whether participants favor high-value items when the procedure does not dictate that they should do so. Thus, our first objective was to test whether a memory advantage for words that were previously trained to have a high (versus a low) reward value persists in later *unrewarded* implicit- and explicit-memory tests (Experiment 1), to test for the generality of reward-value enhancements. Our second objective was to test whether an item-memory advantage for high-value words generalizes if the trained words have to be studied and memorized in a new context (Experiments 2a and 2b).

*Rewarded* memory tests in numerous studies have shown that people are able to prioritize learning of high-value over low-value items, both words and images (Harley, 1965; Tarpy and Glucksberg, 1966; Weiner, 1966; Weiner and Walker, 1966; Loftus and Wickens, 1970; Bjork and Woodward, 1973; Eysenck and Eysenck, 1982; Castel et al., 2002, 2007, 2009; Adcock et al., 2006; Gruber and Otten, 2010; Kuhl et al., 2010; Shohamy and Adcock, 2010; Soderstrom and McCabe, 2011; Wolosin et al., 2012; Watkins and Bloom, unpublished manuscript). For example Castel et al. (2002), showed participants words along with numerical reward values ranging from 1 to 12. Participants were instructed to remember the words with the highest values as best as possible, to maximize the total value of their recalled words. High-value words were recalled more than low-value words. This suggests people were able to flexibly adjust the allocation of cognitive resources during learning to favor items with higher value over those with lower value, and thus maximize earned reward. Assuming a limited resource model, the authors also suggested that if a particular item is allocated more resources, it will be remembered better, but at the expense of the other studied items.

Prioritization effects are not limited to recall; Adcock et al. (2006) demonstrated an enhancement of memory due to reward value using a different explicit memory test: recognition. They presented participants with a high- or low-value reward cue (“$5.00” or “$0.10”) followed by a scene image. Participants were asked to remember the scenes (presented during reward anticipation) and were told that they would earn the respective reward amount if they successfully recognized the images in a memory task the following day. In the recognition test, participants earned the respective reward for recognition hits, and were penalized for false alarms. Hit rates were higher for high- than low-value items. Again, this result demonstrates people’s ability to explicitly prioritize items associated with a higher-value reward over those with a lower-value reward, both during encoding and retrieval.

Such enhancements of memory due to reward value have been found with tests of explicit memory. However, reward value could influence implicit memory in equally powerful ways. That is, reward value might modulate behavior even when the participant is not deliberately trying to retrieve item-values. This would extend the prioritization findings beyond a deliberate encoding/retrieval strategy, and would suggest that in addition, participants may have a cognitive bias toward high-value items. Although it has never been tested directly, some findings are consistent with the hypothesis that higher reward values lead to better implicit memory: Rewards that are presented subliminally can influence behavior (reviewed in Custers and Aarts, 2010). For example, participants respond more quickly (∼20 ms) in simple monetary incentive tasks when the trial is preceded by a high-value reward cue, than if it is preceded by a low-value reward cue (e.g., the participant is presented with the reward cue, and told to press a button once a target appears; Abler et al., 2005; Sescousse et al., 2010; Staudinger et al., 2011). Furthermore Pessiglione et al. (2007), presented participants with coin images of either 1-pound or 1-pence and asked them to squeeze a handgrip to earn the corresponding monetary reward. Coin images were presented either subliminally (for 17 or 50 ms) or supraliminally (100 ms). Participants squeezed the grip harder on the higher-value trials, even when the coin image was not consciously perceived. Hence, consciously and unconsciously processed reward cues can have analogous effects. Subliminally presented higher-value rewards also recruited more attention than lower-value rewards (pupil dilation: Bijleveld et al., 2009) and increased accuracy in arithmetic (Bijleveld et al., 2010). Though none of these studies have directly shown that reward value can enhance implicit memory, they provide at least indirect support for the hypothesis that high-value items could enhance implicit memory.

We also wanted to clearly separate the value-learning phase, which should be rewarded by necessity, from the later memory phase, which should be unrewarded. Our reasoning was as follows: to interpret the prioritization effects, one must consider that participants were instructed to prioritize. The positive prioritization results, therefore, tell us that participants are capable of prioritization. We ask here whether participants have a bias toward better memory for higher-value stimuli in an unrewarded memory test, even when there is no immediate need to favor the encoding of high-value stimuli. By clearly separating the value-learning phase from the memory study phase (Experiments 2a and 2b) and test phase (all experiments here), we can test whether people possess a learning bias universally favoring high over low-reward value items or reward value might interfere with new learning.

Raymond and O’Brien (2009) conducted an experiment along these lines, testing for the non-deliberate effects of reward value on memory (see also Wittmann et al., 2005, 2011), but it is difficult to determine whether their results were driven by implicit- or explicit-memory retrieval. In their value-learning task, stimulus values were learned with repeated experience, and the effects of the learned values on memory were later tested with an unrewarded, modified attentional blink (AB) task. Participants were first presented with pairs of faces and asked to choose one. Faces within-pair differed in their probability of reward (0.20 or 0.80; reward value across pairs was positive, negative, or neutral). Unlike a conventional AB task, Raymond and O’Brien (2009) asked participants not simply to respond when they saw the target image, but instead to indicate whether the target image was an old face from the prior value-learning task, or a new face (i.e., old/new recognition). If a target image were to overcome the AB, it may also be better retrieved in explicit recognition-memory. Higher-value faces were indeed more often recognized as old than lower-value faces, even though, critically, performance in this task was unrewarded. Raymond and O’Brien (2009) concluded that more attentional resources are recruited for stimuli that previously acquired a higher value. Their results also demonstrate a prioritization from a value-learning task where target items are encoded incidentally. However, we suggest that the following interpretations are possible: (a) High-value faces were primed more during value-learning, leading to enhanced implicit memory for higher-value faces during the AB task. Greater priming for the higher-value faces may have led to increases in subjective experiences of familiarity in the recognition-memory test in the AB task. (b) Old/new recognition is a test of explicit memory. Participants may have recognized the high-value faces in the AB task due to episodic recollection (i.e., explicit memory). (c) Recognition in the AB task may have resulted from a combination of implicit- and explicit-memory. Thus, while Raymond and O’Brien’s results provide evidence of a reward-based enhancement of recognition-memory, it is unclear whether this was an enhancement of implicit- or explicit-memory or a mixture both.

In the current study, we first asked if previously learned reward values also enhance item accessibility in an implicit test of memory: lexical decision (Experiment 1). Participants were first presented with words in a two-alternative choice value-learning task, in which they learned, by trial-and-error with feedback, that half of the words led to a high-value reward and half of the words led to a low-value reward (also used by Madan and Spetch, 2012). This value-learning task is similar to previous reward-learning procedures used by Estes and others (e.g., Pubols, 1960; Estes, 1962, 1966, 1972; Humphreys et al., 1968; Allen and Estes, 1972; Medin, 1972a,b) as well several more recent reward-learning studies (e.g., Johnsrude et al., 1999, 2000; Frank et al., 2004, 2006; Bayley et al., 2005; Pessiglione et al., 2006; Valentin and O’Doherty, 2009; Voon et al., 2010; Gradin et al., 2011). Participants were then presented with an unrewarded lexical decision task, in which words from the value-learning task were shown again. Finally, in an unrewarded test, participants were asked to freely recall all the words from the session (value-learning phase and lexical decision). We predicted that explicit memory (free recall) would be enhanced by reward value. We further predicted that implicit memory would be enhanced due to reward value, as measured in the lexical decision task, if reward value enhances memory retrieval even when participants do not deliberately prioritize the retrieval of high-value items over low-value items. If memory is enhanced in both memory tests, we will then ask whether the two effects could have the same underlying cause or not. This will be done by correlating the high-value advantage in lexical decision with the high-value advantage in free recall across participants. If the correlation is large and positive, this would suggest that memory, both implicit and explicit, can be enhanced by reward value through a singular mechanism that globally enhances memory performance. However, implicit- and explicit-memory functions are supported by separable memory systems, both in behavior (e.g., May et al., 2005; Gopie et al., 2011) and in the brain (e.g., Rugg et al., 1998; Schott et al., 2005, 2006). If we instead find that performance in the two memory tasks is uncorrelated or even produce a negative correlation, this would suggest that enhancements of memory due to value are driven by separable reward-based modulations of different kinds of memory.

In a second pair of experiments, we asked if the enhancement of explicit memory due to reward value would persist if items with previously learned reward values were re-studied in a new context. Participants in Experiments 2a and 2b were first given the same value-learning task as in Experiment 1. Following this, participants were asked to study several lists composed of previously learned high- and low-value words, as well as new items, in an unrewarded free-recall task. In this free-recall task, participants had to disregard their memory for items from the value-learning task and instead, confine their memory retrieval to only the most recently studied list (a specific, temporally defined context). Experiments 2a and 2b were identical except that a faster presentation rate was used in Experiment 2b to test whether the results of Experiment 2a could be due to time-consuming processes applied during study, such as deliberate encoding of reward value. Because the list length was short (nine words per list), we expected that total probability of recall might not be a sensitive enough measure; we therefore additionally examined output order and intrusion rates to test whether high- or low-value items were remembered better.

According to the reward-maximization hypothesis, participants devote more resources to learning higher-value items than lower-value items. This should generalize to learning in a new context (determining whether an item was presented within a specific context), which leads to the prediction that free recall will be enhanced for high-value words in Experiments 2a and 2b. According to the value-interference hypothesis, cognitive resources may be diverted to high-value items, and this is at the expense of attention to other related information, including the list context. Thus, the value-interference hypothesis leads to the prediction that free recall will be worse for high-value items, and that high-value items will be intruded more than low-value items (due to failures of list discrimination).

## 2 Experiment 1

### 2.1 Methods

#### 2.1.1 Participants

A total of 99 introductory psychology students at the University of Alberta participated for partial fulfillment of course credit. Five participants were excluded due to machine error. All participants had learned English before the age of six and were comfortable typing. Participants gave written informed consent prior to the study, which was approved by a University of Alberta Research Ethics Board.

#### 2.1.2 Materials

Words were selected from the MRC Psycholinguistic database (Wilson, 1988). Imageability and word frequency were all held at mid-levels and all words had six to seven letters and exactly two syllables. We additionally used the Affective Norms for English Words (ANEW; Bradley and Lang, 1999) to exclude words with moderately arousing, positive, or negative emotional connotations^1^ (e.g., “assault,” “hatred,” and “heaven”) which could interfere with learning reward values (e.g., participants may find it difficult to learn that “hatred” is a high-value word, or that “heaven” is a low-value word). Two words were removed manually as they were deemed by the authors to be emotional in nature, but were not included in ANEW (e.g., “terror,” “regret”). A total of 21 words were excluded this way, and the final word pool consisted of 160 words (Table 1 reports word pool properties).

**Table 1:** **Word pool statistics, as obtained from the MRC Psycholinguistic database (Wilson, 1988)**.

	Concreteness	Imageability	Word frequency	Word length	Number of syllables
Mean	439	467	22	6.46	2
SD	99	80	12	0.50	0
Min	243	248	7	6	2
Max	580	578	52	7	2

For the lexical decision phase, 160 pronounceable non-words were generated with the LINGUA non-word generator (Westbury et al., 2007), using a pre-compiled word frequency dictionary (Shaoul and Westbury, 2006). To match the length of the non-words to the words, we generated 87 six-letter and 73 seven-letter non-words.

#### 2.1.3 Procedure

Prior to the experiment, participants were informed that the experiment was a “word choice task,” and that they would receive a payment proportional to the total points earned in the value-learning task of the experiment, in addition to their partial course credit.

The experiment consisted of a sequence of four tasks: value-learning, lexical decision, free recall, and a value-judgment task. Participants were not provided with details of the subsequent task until the current task was completed.

##### 2.1.3.1 Value learning

Participants were shown two words on the computer screen simultaneously. Words were selected at random from our word pool of 160 words. Participants were to choose one of the two words in each choice set by pressing the “Z” or “/” key of a computer keyboard to choose the word presented on the left or right side of the computer screen, respectively.

For each participant, 36 words were randomly selected from the word pool, and each word was randomly assigned to one of two reward values: 1 or 10 points (low- or high-value, respectively). Trial choices were pseudorandomly generated, with each word used one time per choice set, but each set always consisted of one high- and one low-value word. This constraint was not revealed to the participant. After each choice, the participant saw the reward in the center of the screen for 2000 ms; if they chose a high-value word, an image of a pile of coins was presented; if they chose a low-value word, an image of a penny was presented. The participant’s current point balance was continually presented at the bottom of the screen throughout the duration of the value-learning task. There was no time limit on the choices and participants were given a 1000-ms delay before the next choice.

Training consisted of 18 choice sets per block for 13 blocks. At the end of the session, participants were paid $1.00 for every 500 points earned during the value-learning task, rounded up to the nearest 25-cent amount. Participants earned between $3.25 and $5.00 in this task.

##### 2.1.3.2 Lexical decision

An additional 18 words, selected at random from the same pool as the trained words, were included as new words. Participants were asked to judge the lexical status of 108 items: 36 trained words, 18 new words, and 54 non-words. Each item was presented for up to 10,000 ms, and the participant pressed either “Z” on the computer keyboard to indicate that the item was a proper English word, or “/” to indicate that the item was a not a word. A fixation cross (“+”) was presented for 1000 ms to separate each decision prompt.

The 108 items were preceded by eight practice items (four words/four non-words) to attenuate a possible recency effect over the last words from the preceding value-learning task.

##### 2.1.3.3 Free recall

In a final free-recall task, participants were given 5 min. to recall all of the words they could remember from the study, in any order. Participants were asked to type out their responses, terminated with the ENTER key. After each response, a blank screen was presented for 500 ms. Repeated recalls of the same words were ignored.

##### 2.1.3.4 Value judgment

To measure participants’ explicit memory of the reward values for each item, we included a value-judgment task following free recall. At the end of the experiment, participants were presented with each of the words previously shown in the value-learning task, one at a time, and asked to judge how many points each word had been worth in the initial value-learning task. Participants were told to press the “Z” key if they thought the word was worth 1 point, or “/” for 10 points.

#### 2.1.4 Data analysis

Effects were considered significant based on an alpha level of 0.05. For response time analyses, only correct responses were analyzed. Response time analyses were conducted on the within-subject median response time for each condition.

For lexical decision, only responses made between 200 ms and the individual participant’s mean plus 3 SD were included in the analysis (0.61% trials excluded).

### 2.2 Results and discussion

**Figure 1:**
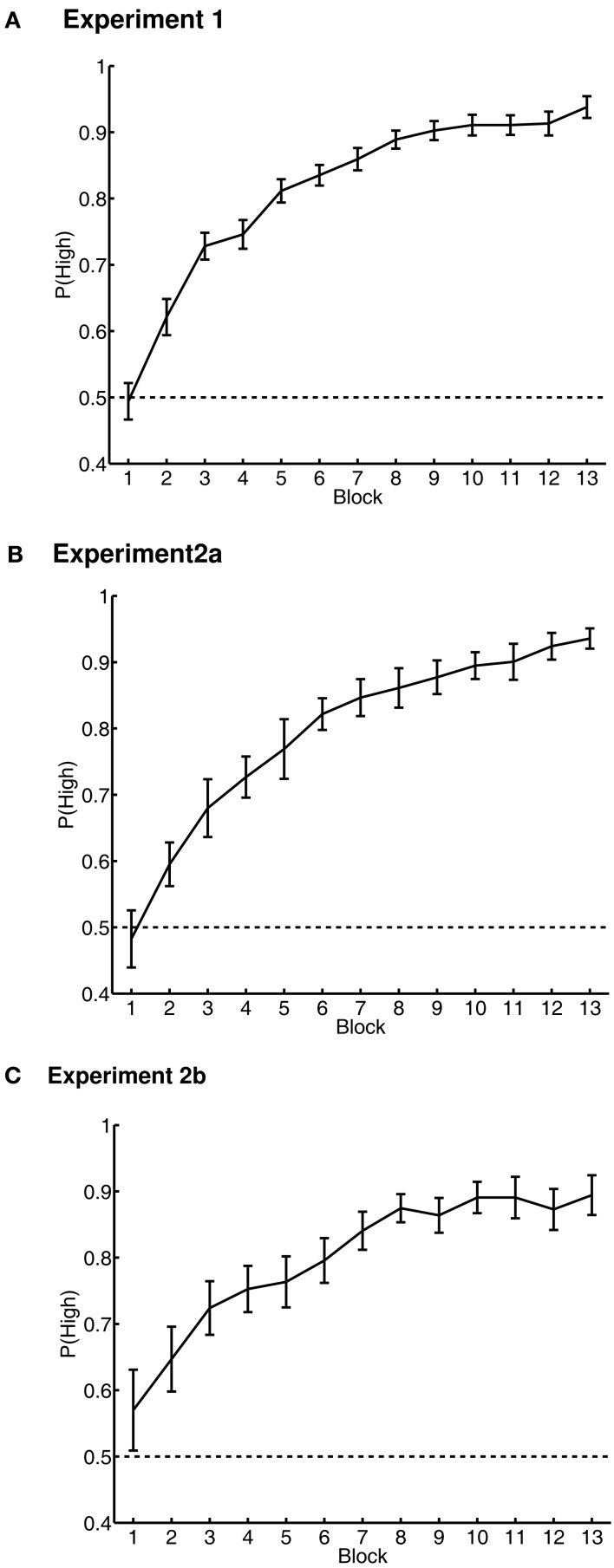
**Value-learning task results in Experiments 1 (A), 2a (B), and 2b (C)**. Performance is shown as the probability of choosing the high-value word over the low-value word in each of the learning blocks 1 to 13. Chance probability of choosing the high-value word is indicated by the dashed line. Error bars are 95% confidence intervals around the mean, corrected for inter-individual differences (Loftus and Masson, 1994).

Accuracy in the value-learning task was measured as the proportion of trials on which the participant chose the high-value word. This measure began at chance, as the participant could not know which was the high-value word. In the last block of the value-learning task, accuracy was significantly greater than chance and near ceiling [*M* ± 0.95 CI = 0.94 ± 0.02 correct; *t*(93) = 37.34, *p* < 0.001] (Figure 1A).

**Figure 2:**
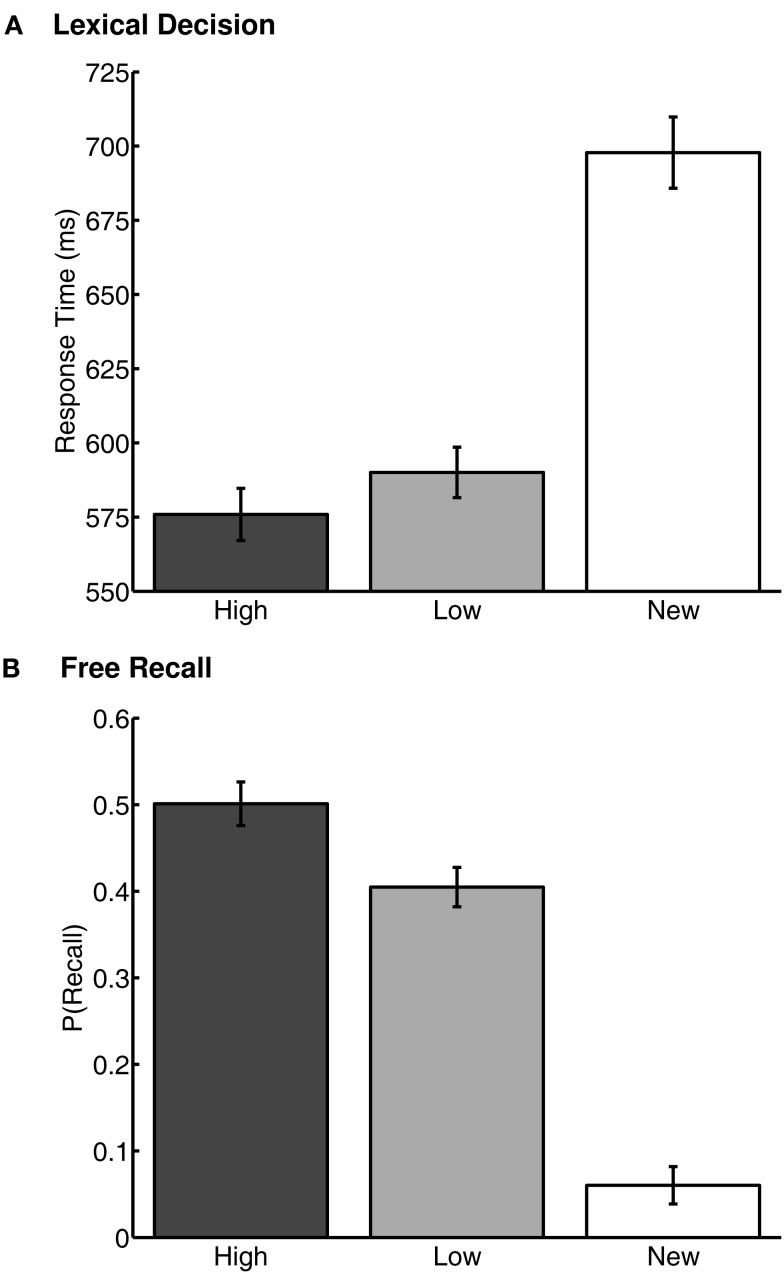
**Performance in the memory tasks in Experiment 1. (A)** Response times from the lexical decision task. **(B)** Proportion of total words recalled from the free recall task. “High” and “Low” represent the high- and low-value words, respectively. “New” represents words first used in the lexical decision task, that were not present in the value-learning task. Error bars are 95% confidence intervals, corrected for inter-individual differences (Loftus and Masson, 1994).

Lexical decision was significantly more accurate for the previously rewarded old words than for the untrained, new words [*t*(93) = 6.94, *p* < 0.001; old words: 0.99 ± 0.03 correct; new words: 0.95 ± 0.14 correct]. Participants also identified the old words significantly faster than the new words [*t*(93) = 12.77, *p* < 0.001, *M*(new) = 708 ± 12 ms; *M*(old) = 591 ± 8 ms]. There was no difference between accuracy for high- and low-value words [*t*(93) = 0.00, *p* > 0.1; high value: 0.99 ± 0.04 correct; low-value: 0.99 ± 0.04 correct]. Importantly, high-value words were identified significantly faster than low-value words [*t*(93) = 2.42, *p* < 0.05; *M*(high) = 584 ± 9 ms; *M*(low) = 599 ± 8 ms] (Figure 2A). Thus, trained words were primed, and high-value words were primed more than low-value words, a novel finding that suggests that reward value can influence implicit memory.

Probability of free recall (Figure 2B) was greater for high-value words than low-value words [*t*(93) = 4.40, *p* < 0.001; *M*(high) = 0.50 ± 0.04; *M*(low) = 0.40 ± 0.03]. “New” words (from the lexical decision task) were also recalled, but far less often than the previously rewarded words [*t*(93) = 23.80, *p* < 0.001; *M*(new) = 0.06 ± 0.01; *M*(old) = 0.45 ± 0.03]. Thus, value also influenced explicit memory retrieval, replicating prior findings.

We next asked if the memory effects of value depended on the level of performance during value training. However, the asymptotic accuracy in the value-learning task (averaged over the last four trials) did not correlate significantly with the value effects on both memory tests [both *p*’s > 0.1].

In the value-judgment task, participants rated the value of the previously rewarded words much better than chance [*M* = 0.87 ± 0.03 correct; *t*(93) = 25.65, *p* < 0.001]. The accuracy of judgments was similar for high-value words [*M*(high) = 0.88 ± 0.03 correct] and low-value words [*M*(low) = 0.87 ± 0.03 correct; *t*(93) = 1.16, *p* > 0.1]. That is, participants had substantial, though not perfect, explicit memory for the value of both high and low-value words. In the value-learning task, because all responses were a choice between a high- and a low-value item, those responses cannot be used to determine whether high and low values were learned to the same level. In the value-judgment task, items were judged individually; thus, the near-equivalence of value judgments of high- and low-value items suggests that participants learned the values of high- and low-value words equally well. This rules out the possibility that participants simply remembered the high-value words better because they performed the value-learning task better for high- than low-value items. It could further be argued that the value judgments for both types of items could have been based on memory for high-value items alone: A participant then would decide to judge a high-value item as “high” based on their memory for that item’s value, but to judge all items for which they had no such memory as a “low” item. That is, value judgments would be made on a single value dimension. If only the strength of memory for high-value items was used to make judgments along this dimension, high-value words could be correctly classified as high (those with sufficient high-value item-memory strength), low-value words could be correctly classified as low (those with insufficient high-value memory strength), and high-value words could be incorrectly classified as low (those with insufficient high-value memory strength). However, low-value words could not be incorrectly classified as high-value words this way. As reported, we did observe such errors in 13.2% of the low-value words. Note also that the probability of judging a low item as high was quite close to the probability of judging a high item as low (12.5%). Thus, regardless whether participants are basing their choices on a singular value dimension, they are doing so with the same accuracy for low as for high items. This suggests that the quality of memory (i.e., variance in memory strength for both word types along a value dimension) is equivalent for both types of words.

One plausible explanation of our results is that, instead of value, our effects on memory are due to choice behavior: the more often a participant chose an item during value-learning, the more they remembered that item in the later memory tests (see Weber and Johnson, 2006). Since choice frequency and value are highly confounded (i.e., the task *requires* choosing high over low-value items), a combined correlation spanning all items would not be possible either. As an alternative, we calculated choice frequency as the mean number of times a participant chose a high-value item, across all 13 blocks of the value-learning task, minus the mean number of times they chose a low-value item: DiffCF = *mean*[*choice frequency* (*H*)] − *mean*[*choice frequency* (*L*)]. DiffCF thus measures a participant’s bias toward choosing high- over low-value words. DiffCF is, of course, expected to be highly correlated with accuracy, since participants are indeed asked to choose high items and to avoid low items. Confirming this, the correlation between participants’ accuracy in the value-training task and the DiffCF measures was highly significant [ρ(93) = 0.48, *p* < 0.001]. To rule out that choice frequency significantly co-varied with our effects of value on implicit- and explicit-memory, we then correlated DiffCF with: (a) the effect of value on lexical decision performance (the normalized difference in response times due to reward value: DiffLD = [*RT*(*low*) − *RT*(*high*)]/0.5[*RT*(*low*) + *RT*(*high*)]; (b) the effect of value on free-recall performance, DiffFR = proportion of recalled high-value words, divided by the total number of words recalled. DiffCF correlated with neither the effect of reward value on implicit memory, nor the effect of reward value on explicit memory [lexical decision: ρ(93) = 0.075, *p* > 0.1; free recall: ρ(93) = 0.074, *p* > 0.1]. Thus, the bias to choose high over low-value words in the value-learning task did not account for the effects of reward value on implicit- or explicit-memory. This is consistent with Madan and Spetch (2012), who also ruled out choice frequency as a possible determining factor of subsequent memory with a similar training procedure.

**Figure 3:**
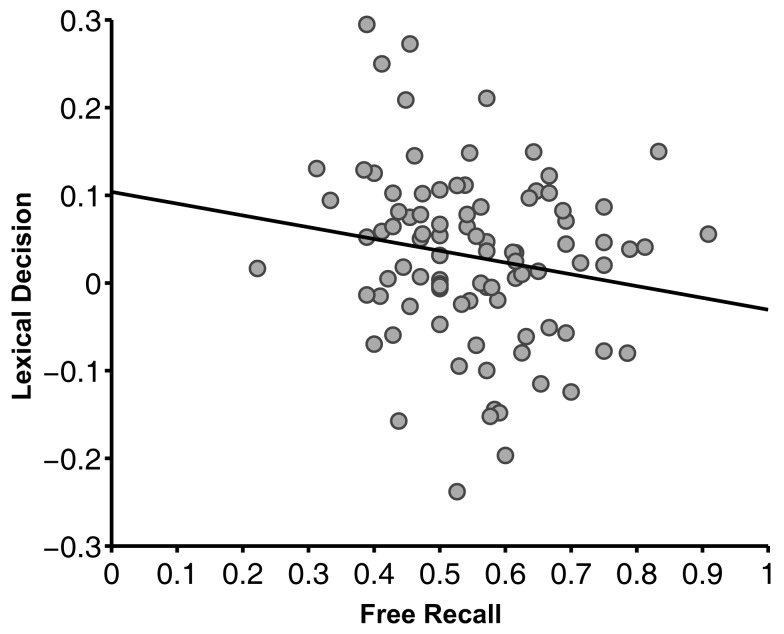
**Correlation between lexical decision and free recall tasks in Experiment 1 [ρ(93) = −0.20, *p* < 0.05]**. The lexical decision measure was the facilitation of high-value words compared to low-value words (difference in response time) divided by the participants’ average response time. The free recall measure was the proportion of recalled words that were high value, divided by the total number of words recalled from the value-learning task. Each dot represents an individual participant.

Next, we asked whether the effects of reward value on our two memory tests were related, explaining common variance across participants, or unrelated, explaining different subject variability. Participants who demonstrated greater value-based facilitation in lexical decision had *less* value-based facilitation in free recall [ρ(93) = −0.20, *p* < 0.05] (Figure 3). The fact that a positive correlation was not observed suggests that there are at least two partly dissociable mechanisms by which value can influence memory.

Because lexical decision always preceded final free recall, we were concerned that the negative correlation between the two value-based facilitation effects on memory could be due to the influence of lexical decision on free recall. If a word had a long response time in lexical decision, perhaps that would correspond to increased encoding of the word; a poor lexical decision response might then turn into an increased probability of free recall. We tested for this kind of effect with within-subjects analyses. We compared lexical decision response times for words that were or were not free recalled, separately for high- and low-value words. Lexical decision response times were not significantly different between later recalled and later not recalled words [high value: *t*(93) = 1.13, *p* > 0.1, Cohen’s *d* = 0.07; low-value: *t*(93) = 0.60, *p* > 0.1, *d* = 0.04]. Thus, the effect of an item’s value on explicit memory is not explainable by its effect on implicit memory, or vice versa, and this rules out explanations due to the fixed task order, i.e., the possibility that the negative correlation between value-based facilitation effects was merely due to further encoding during lexical decision. Instead, we found no relationship between lexical decision time for an item and its later recall probability, in line with our previous interpretation of the between-subjects correlations: the enhancements in the two tasks were driven by different mechanisms.

We had not expected the negative correlation between the effects of value on implicit- and explicit-memory. In an attempt to derive an explanation *post hoc*, we took a closer look at our data. Perhaps the observed negative correlation between implicit- and explicit-memory had been driven by differences in participants’ learning strategies in the value-learning task (even though, as reported above, the asymptotic accuracy in the value-learning task did not correlate significantly with the value effects on the two memory tests). We speculated that participants who learned values earlier may be the ones who showed greater effects of value on implicit memory, because they would have had a larger number of trials on which they knew the correct values. In contrast, participant who took longer to learn presumably found the value-learning task more challenging early on; for these participants, value may have been used more as a deliberate retrieval cue in later explicit memory. For this purpose, we measured how long it took for participants to reach an 80% accuracy criterion in the value-training task (i.e., trials-to-criterion, TTC: choosing the high-value item on 80% of all trials within a block). We then correlated TTC with reward effects on the lexical decision task (DiffLD) and with reward effects on free recall (DiffFR). Note that 6 participants never reached the 80% accuracy criterion; for these participants, the TTC was set to 14, i.e., one greater than the actual number of trial blocks presented in the value-learning task. This correction to the TTC measure served to denote that these participants required more learning trials to reach 80% accuracy. In line with our reasoning, we found that participants who reached the learning criterion *earlier* exhibited stronger implicit memory effects due to reward value (i.e., greater priming in the lexical decision task, DiffLD) [ρ(93) = − 0.22, *p* < 0.05]. This may provide evidence that participants who had learned items values earlier (fewer trials-to-criterion) had more trials on which to accumulate value learning, which then enhanced implicit memory for high-value items. Complementing this result, we found that participants who took *longer* to reach the learning criterion exhibited stronger explicit memory effects due to reward value (i.e., greater difference in recall probabilities in the free-recall task, DiffFR) [ρ(93) = 0.24, *p* < 0.05]. This is consistent with the idea that participants for whom value learning was initially more challenging may have used value more as an explicit memory cue in free recall. Further, when controlling for trials-to-criterion in a partial correlation analysis, the negative correlation between the effects of value on lexical decision and free recall was no longer negative, and far from significant [ρ*_p_*(93) = 0.040, *p* > 0.1]. Although our specific interpretation is *post hoc* and should be considered with caution, the results of this analysis at least suggest that the way people learned the values initially mediated the mutually exclusive effects of value on implicit- and explicit-memory.

### 2.3 Summary

Experiment 1 revealed that high-value words were subsequently remembered better than low-value words in *both* implicit- and explicit-unrewarded memory tests. The effect of value on memory in these two memory tasks was slightly negatively correlated, suggesting the presence of at least two mechanisms mediating the memory enhancement by reward value, rather a global enhancement of memory due reward value. Different initial value-learning strategies may have contributed to this negative correlation.

The enhancement of implicit memory by value (i.e., an accessibility bias for high-value items), is a finding without direct previous evidence. Although the influence of reward value on response time in our lexical decision task was relatively small (∼15 ms), this is consistent with studies that presented a reward cue in monetary incentive tasks and found that reward value facilitated response time by ∼20 ms (Abler et al., 2005; Sescousse et al., 2010; Staudinger et al., 2011). Furthermore, nearly all prior studies demonstrating the reward-based enhancement of memory used procedures that led to the deliberate prioritization of encoding due to reward value. Here we used a procedure where participants incrementally learned values and found the enhancement of both implicit- and explicit-memory due to reward value. Because the memory tests were unrewarded, and no prioritization instructions were given, this suggests that not only can participants prioritize when asked to, but they exhibit a bias to learn high-value words better than low-value words. Such a bias may serve them well in naturalistic situations, in which items usually retain their value.

## 3 Experiments 2a and 2b

We next asked whether the high-value item advantage observed following training in Experiment 1 would extend to a new learning situation involving the reward-value-trained items. Having established that the value-learning procedure in Experiment 1 can enhance explicit memory due to reward value, we used the same procedure to test for effects of reward value on new learning involving value-trained words in a different context. Following training as in Experiment 1 and a distractor task, we had participants learn word lists consisting of trained words and untrained words. In a study/test procedure, participants viewed each new list, followed by delayed free recall.

As in Experiment 1, our dependent measure in the free-recall task was the proportion of words recalled of each word type (high value, low value, or new). However, proportion of recalls is a rather coarse measure of memory, as it collapses across all responses given by a participant on a list. Apart from being a test of item retrievability, free recall is also a test of associations between items and a specific list context. In other words, words output earlier in free recall represent the items that are easier to retrieve and also have the strongest associations with the current target-list context (e.g., Bjork and Whitten, 1974; Crowder, 1976; Raaijmakers and Shiffrin, 1981; Howard and Kahana, 1999; Brown et al., 2007). Likewise, late in the recall sequence, responses are more likely guesses. Thus, in addition to recall accuracy, we tested if any word type (high value, low value, and new) was recalled significantly earlier or later than any other word type.

We considered two hypotheses: our reward-maximization hypothesis led to the prediction that participants will recall more high-value than low-value words, due to prioritized study of the words that had the high values previously, similar to previous studies finding an enhancement of memory due to reward value when rewards are earned for successful memory performance (e.g., Castel et al., 2002; Adcock et al., 2006). This hypothesis is also suggested by investigations of the effects of emotional arousal on memory, such as Hadley and MacKay’s (2006) priority-binding hypothesis which proposes an enhancement of contextual binding due to arousal (also see Siddiqui and Unsworth, 2011). Alternatively, our value-interference hypothesis led to the opposite prediction: Words with a previously acquired high reward value will be harder to learn and remember in a new context than words with a low-reward value if higher values direct attention toward the high-value items themselves, but away from other pertinent information. This hypothesis also suggests that for the high-value words, participants will find it difficult to constrain their memory retrieval processes to just the list context of the most recently studied list and will instead erroneously recall more high-value words than low-value words. This hypothesis is based on studies finding an impairment of new associative memories between cues that had previously been predictive of emotionally arousing information (Mather and Knight, 2008; Novak and Mather, 2009; Sakaki et al., 2011; Nashiro et al., 2012). For example, Mather and Knight (2008), found that participants had more difficulty learning new associations between sounds/faces and nearby presented digits (and other contextual information), if the sounds/faces had initially been paired with negative images, an effect that did not occur when they had been paired initially with neutral images. This suggests, emotional arousal may have interfered with participants’ ability to learn subsequent associations. Furthermore, Novak and Mather (2009) had participants learn screen locations for neutral and negative images. When locations for individual pictures remained the same over several study–test cycles, participants made more location memory errors for emotional than neutral images in later cycles. Thus, an initial incorrect association between an emotional picture and a location may have led to more interference with learning the correct association than an initial incorrect association for a neutral picture. Moreover, when the locations for individual pictures were switched after three cycles, participants were worse at updating their memory with the new locations for negative images as opposed to neutral images. Together, these findings imply that emotional items are more affected by proactive interference from previous experience with the items, which may present as impaired learning of new associations with such items.

We conducted two variants of this experiment; Experiment 2b had a faster list presentation rate, to further test whether possible effects of previous reward value on new list learning are driven by a time-consuming strategy applied during study (i.e., value-based prioritization of encoding or retrieval).

### 3.1 Methods

#### 3.1.1 Participants

A total of 72 introductory psychology students at the University of Alberta participated for partial fulfillment of course credit. All participants had normal or corrected-to-normal vision, learned English before the age of six, and were comfortable typing. Participants gave written informed consent prior to the study, which was approved by a University of Alberta Research Ethics Board. Participants never participated in more than one of Experiment 1, 2a, and 2b. Experiment 2a had 40 participants; Experiment 2b had 32 participants.

#### 3.1.2 Materials

The same materials as used in the training phase of Experiment 1 were used in both Experiments 2a and 2b.

Six maze puzzles were generated for the distractor task using an online maze generator (http://www.hereandabove.com/maze/mazeorig.form.html). Mazes were made using the generator’s default settings.

#### 3.1.3 Procedure

The experiment consisted of three tasks performed in a fixed sequence: value-learning, maze distractor, and study/test free recall of six nine-word lists. Participants were not provided with details of the subsequent task until the current task was completed.

##### 3.1.3.1 Value learning

The procedure was the same as in Experiment 1.

##### 3.1.3.2 Maze distractor

To reduce the very high level of proactive interference from the value-training phase on the free-recall phase, we included a non-verbal distractor task following value training. Participants were given 5 min. to complete pencil-and-paper mazes. When participants finished one maze, they were provided with another maze. This procedure was repeated until the 5 min. had elapsed, at which point the maze was removed and the participant advanced to the study/test free-recall task. On average, participants completed 2–3 mazes within the 5 min.

##### 3.1.3.3 Study/test free recall

Participants were told to study each list of words and that their memory for the list would be tested, but that they would not earn any reward in this phase. Participants first studied one practice list of 9 words from the word pool that were excluded from analyses, and 6 experimental lists of 9 words each: 3 high-value words from the value-learning task, 3 low-value words from the value-learning task, and 3 new words (random order of presentation in each list).

Each word was presented for 1800 or 800 ms (Experiment 2a and 2b, respectively), after which the screen was cleared for 200 ms. After being presented with all 9 words, participants were given a distractor task that consisted of four equations in the form of A + B + C = ___, where A, B, and C were randomly selected digits between 2 and 8. Each equation remained in the center of the screen for 5000 ms. The participant was asked to type the correct answer during this fixed interval, after which the screen was cleared for 200 ms.

After the distractor, participants were given 1 min. to recall as many of the words from the list that they could (i.e., free recall). Participants were asked to type out their responses. After each response, a blank screen was presented for 500 ms. Participants were allowed to pause prior to the presentation of the next list. This procedure (list encoding, math distractor, and list free recall) was repeated for all 6 lists.

#### 3.1.4 Data analysis

Effects were considered significant based on an alpha level of 0.05. ANOVAs are reported with Greenhouse–Geisser correction for non-sphericity where appropriate and *post hoc* pairwise comparisons are Bonferroni-corrected.

Because the value-learning task consisted of 13 trials, which means participants had 13 presentations of each stimulus, we expected there to be a large amount of proactive interference. As participants could not advance to the next list until the full minute expired, we expected later responses to include a high level of guesses. However, we were also concerned that some participants may not have understood that they were to confine their responses to the very last list presented. Therefore, to identify such non-compliant participants, we screened out participants who had extremely low accuracy *early* in the output sequences.

We calculated the average proportion of correct recalls within the first four responses to ensure that participants included in the analysis attempted to recall items from the most recent list (i.e., that they followed instructions). We found that most participants responded with three correct recalls in their first four responses [Experiment 2a: *M* = 3.46; Experiment 2b: *M* = 3.32]. However, five participants produced an average of one or fewer correct recalls in their first four responses [Experiment 2a: two participants; Experiment 2b: three participants] and were excluded from further analyses. Excluding these participants, the number of correct recalls in the first four responses did not substantially change the mean correct recalls within the first four recalls of the entire samples [Experiment 2a: *M* = 3.50; Experiment 2b: *M* = 3.35]. Similarly, total number of correct recalls did not change much [Experiment 2a, total sample: *M* = 9.45; Experiment 2a, excluding 2 participants: *M* = 9.35; Experiment 2b, total sample: *M* = 9.91; Experiment 2b, excluding 3 participants: *M* = 9.39]. Thus, exclusion of these five participants did not substantially change the observed recall patterns. For the remaining participants, analyses were carried out after removing extra-experimental intrusions and within-list repetitions.

### 3.2 Results and discussion

#### 3.2.1 Value learning

The value-training data resembled the data in Experiment 1 (Figures 1B,C). Performance again began near chance, and improved across blocks; in the last block (block 13), accuracy was significantly greater than chance [Experiment 2a: *t*(37) = 31.16, *p* < 0.001, *M* = 0.94 ± 0.03 correct; Experiment 2b: *t*(30) = 12.83, *p* < 0.001, *M* = 0.89 ± 0.06 correct].

#### 3.2.2 Study/test free recall

##### 3.2.2.1 Proportion of words recalled

**Figure 4:**
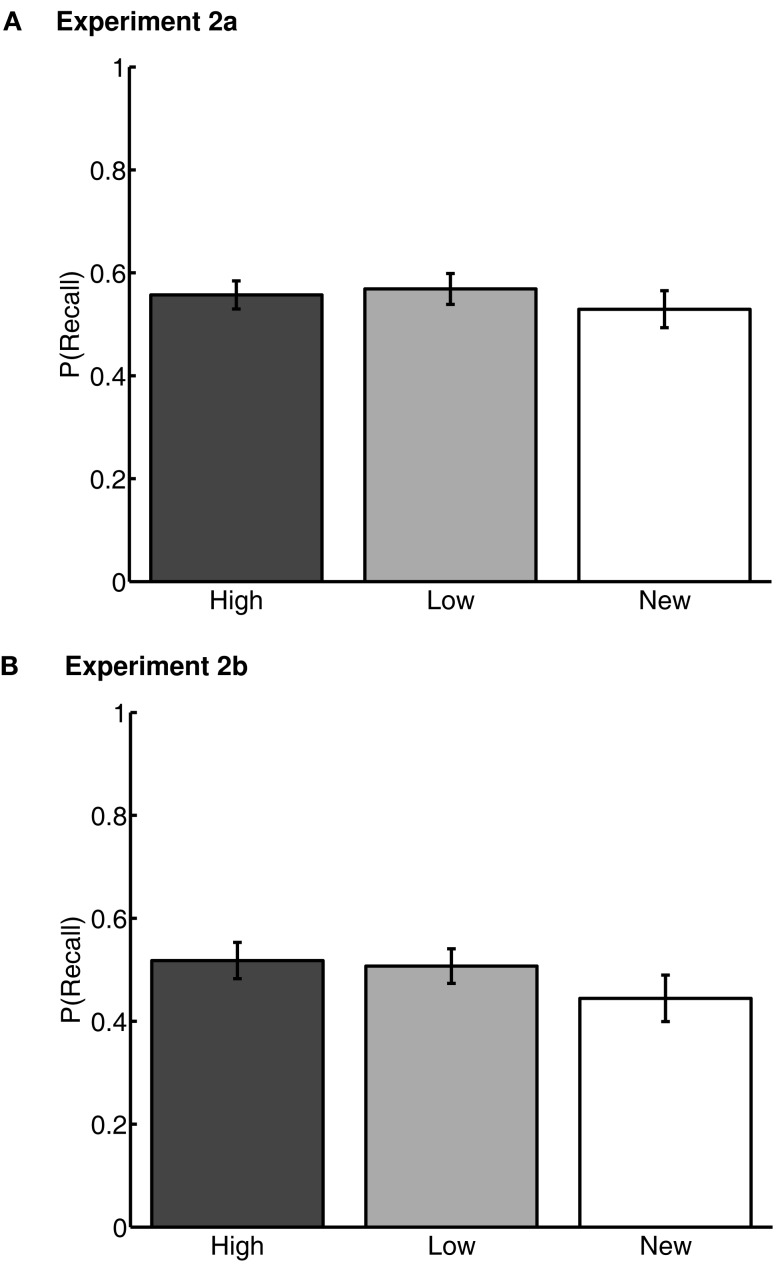
**Correct recall rates for Experiments 2a (A) and 2b (B)**. “High” and “Low” represent high- and low-value words, respectively, from the value-learning task. “New” represents words that were not present in the value-learning task, but only in study-test free recall. Error bars are 95% confidence intervals, corrected for inter-individual differences (Loftus and Masson, 1994).

In each of Experiments 2a and 2b, we conducted repeated-measures ANOVAs on Word Type (high value, low value, and new) on the proportion of words recalled. Proportion recalled was defined as the average number of correct words recalled of each word type across lists, divided by 3 (the number of words of each type in each list). The main effects of Word Type were not significant in either experiment [Experiment 2a: *F*(2,67) = 1.08, *p* > 0.1, $\eta_{p}^{2}$ = .03, *M*(high) = 0.56 ± 0.04, *M*(low) = 0.57 ± 0.04, *M*(new) = 0.53 ± 0.05; Experiment 2b: *F*(2,52) = 2.74, *p* > 0.1, $\eta_{p}^{2}$ = .08, *M*(high) = 0.52 ± 0.05, *M*(low) = 0.51 ± 0.06, *M*(new) = 0.44 ± 0.06] (Figure 4). The lack of a difference in recall rates of high-value and low-value words suggests that, by this measure, effects of previously learned value on memory had been neutralized.

##### 3.2.2.2 Output order

**Figure 5:**
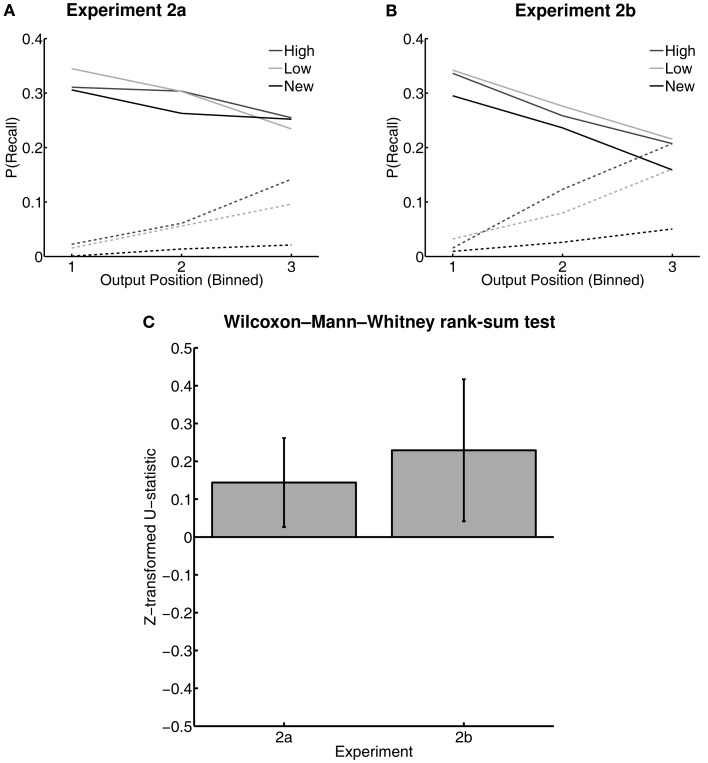
**Output positions in the free recall task of Experiments 2a and 2b**. Probability of recall of each word type for vincentized output position bins in the free recall task of Experiments 2a **(A)** and 2b **(B)**. “High” and “Low” represent high- and low-value words, respectively, from the value-learning task. “New” represents words first that were not present in the value-learning task. Solid lines and markers represent correct responses; dashed lines with hollow markers represent intrusion responses. Error bars were omitted for visual clarity. **(C)** Plots of the *Z*-transformed *U*-statistics comparing median output positions for high- versus low-value words in both Experiments 2a and 2b. Larger values represent later output positions. Error bars are 95% confidence intervals.

To analyze output order for each Word Type (high value, low value, and new) directly, we borrowed the logic of the Wilcoxon–Mann–Whitney rank-sum test on the output positions of each word type to derive a measure of differences in median output position for each Word Type (as suggested by Hubert and Levin, 1978). For each list, for each pairwise Word Type comparison, the *U*-statistic was *Z*-transformed and then averaged across lists to obtain a measure for each participant. Because these values were already *Z*-scores, they were then compared with a *t*-test against zero for each comparison between Word Types. Participants with no recalls of a given Word Type in two or more lists were excluded from this analyses as they did not contribute additional information to this analysis (leaving *N* = 35 and 29 in Experiments 2a and 2b, respectively). In both experiments, low-value words had significantly earlier median output positions than high-value words [Experiment 2a: *mean*(*Z_U_*) = 0.14, *t*(34) = 2.40, *p* < 0.05, *d* = 0.46; Experiment 2b: *mean*(*Z_U_*) = 0.23, *t*(28) = 2.35, *p* < 0.05, *d* = 0.42] (Figure 5), suggesting that low-value words were easier to recall. High- and low-value words did not differ significantly in median output position relative to new words [all *p*’s > 0.1].

##### 3.2.2.3 Intrusions

In Experiment 1, we found that high-value items were more retrievable in a final free-recall test. Therefore, participants might be more likely to guess high- than low-value words in free recall of the 9-word lists in Experiments 2a and 2b. If list discrimination were enhanced for high-value words, following from the reward-maximization hypothesis, participants should make fewer intrusions of high-value words than low-value words. However, if participants had a more difficult time determining whether high-value items belonged to the current list, following from the value-interference hypothesis, then we should instead find more intrusion responses for high-value words than for low-value words. As words were not re-used from one list to the next, intrusions were defined as words that were not on the target (most recently studied) list. Intrusions could come from the training or else from prior free-recall study lists.

**Figure 6:**
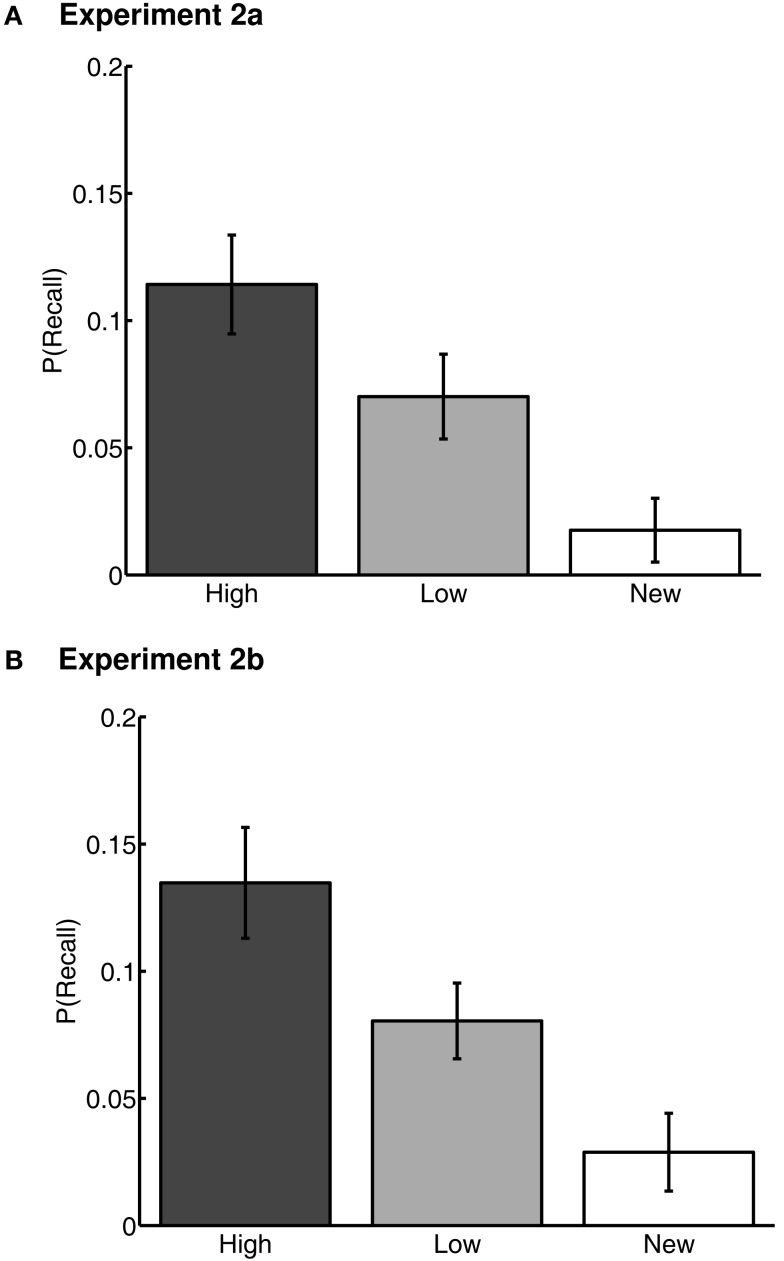
**Intrusion rates during free recall in Experiments 2a (A) and 2b (B)**. “High” and “Low” represent high- and low-value words, respectively, from the value-learning task. “New” represents words first that were not present in the value-learning task. Error bars are 95% confidence intervals, corrected for inter-individual differences (Loftus and Masson, 1994).

Repeated-measures ANOVAs were conducted on intrusion rates. The measure was the proportion of all responses (excluding extra-experimental intrusions and repetitions) on a given list that were intrusions of each word type, averaged across lists. Participants with fewer than three intrusions in total were excluded from only this analysis as they provided an insufficient number of data points (leaving *N* = 24 and 22 included participants in Experiments 2a and 2b, respectively). The main effect of Word Type was significant in both experiments [Experiment 2a: *F*(2,3,4) = 22.11, *p* < 0.001, $\eta_{p}^{2}$ = .51, *M*(high) = 0.11 ± 0.03, *M*(low) = 0.070 ± 0.024, *M*(new) = 0.018 ± 0.010; Experiment 2b: *F*(2,3,4) = 23.14, *p* < 0.001, $\eta_{p}^{2}$ = .50, *M*(high) = 0.13 ± 0.04, *M*(low) = 0.081 ± 0.017, *M*(new) = 0.029 ± 0.018] (Figures 6A,B). High-value words were more likely to intrude than both low-value words [Experiment 2a: *t*(23) = 2.55, *p* < 0.05; Experiment 2b: *t*(21) = 3.12, *p* < 0.01] and new words [Experiment 2a: *t*(23) = 6.73, *p* < 0.001; Experiment 2b: (21) = 6.00, *p* < 0.001]. Low-value words were also intruded more than new words [Experiment 2a: *t*(23) = 4.64, *p* < 0.001; Experiment 2b: *t*(21) = 4.84, *p* < 0.001]. This result also supports the value-interference hypothesis, which suggested that high-value words are harder to place uniquely within the target list (i.e., contextual binding). Moreover, the small advantage of high-value words over low-value words following training in Experiment 1 (ratio of ∼5:4) evolved into a much larger ratio (∼3:2) in the intrusion rates of Experiments 2a and 2b. If guessing were purely based on better retrievability caused by high value and measured by final free recall in Experiment 1, we would have expected the same ratio for intrusion rates, as the words would inherit the same distribution from the final free-recall data. The fact that the ratio is exaggerated for intrusions here suggests that this measure is influenced by more than just item retrievability; we suggest that high-value words were not only sampled more often as candidate responses, but were also screened less well, and thus, were more likely to be recalled in error.

### 3.3 Summary

In both Experiment 2a and 2b, previously trained words were correctly recalled at equal rates, regardless of reward value; thus, the advantages we saw for high-value words in Experiment 1 did not carry forward to a situation in which participants had to relearn subsets of trained words and link them to a specific, new list context. Moreover, high-value words were output *later* in the recall sequence than low-value items, and were more likely to be retrieved erroneously (reflecting proactive interference). This suggests that they were more weakly linked to the current-list context, and were more difficult to accurately screen based on recent-list membership. This pattern of findings held both for a slower presentation rate (2 s/word in Experiment 2a) and for a faster presentation rate (1 s/word in Experiment 2b), and the magnitudes of the output-order and intrusion-rate effects were similar between experiments (Figures 5 and 6). This suggests that the effects of value unlikely result from a deliberate, effortful, and time-consuming process during study (e.g., participants deliberately diverting attention toward the low-value words). It is more plausible that the difference between performance on high- and low-value words was due to persisting effects of reward value on memory from the value-learning task. This would make current-list membership more confusable for high-value items and screening candidate responses more difficult for high- than for low-value items.

## 4 General Discussion

In two studies, we investigated the influence of previously trained reward value on unrewarded tests of memory. In Experiment 1, implicit memory (facilitated access in lexical decision) was enhanced by reward value, in addition to enhanced explicit memory due to reward value (probability of recall in final free recall). These two memory enhancements were negatively correlated across participants, suggesting the presence of at least two mechanisms whereby reward value can influence memory. In Experiments 2a and 2b, we found that previously learned reward values can cause problems for contextual binding, when trained items needed to be tied to a new, specific context (namely, belonging to the most recent list). Low-value items were produced earlier in recall than high-value items, and high-value items intruded more often, suggesting that they were not effectively screened as belonging to the wrong list. The interactions between reward value and memory are thus multifaceted, with implicit- and explicit-memory being enhanced due to reward value through different mechanisms (Experiment 1), and reward value leading to impaired memory for contextual information (Experiments 2a and 2b).

### 4.1 Influence of reward value on implicit- and explicit-memory

In Experiment 1, we observed an enhancement of both explicit *and* implicit memory due to reward value. Since value enhanced both our memory measures, one may have expected that value-learning globally enhanced all kinds of learning of high-value items. For instance, enhanced memory could have been driven by value solely through the recruitment of additional attentional resources during the value-learning task: If participants paid more attention to the high-value items than the low-value items during this first phase, high-value items may then be more primed in lexical decision and more retrievable in free recall. Both enhancements would then have originated from a single, global, value-learning mechanism resulting in a high, positive correlation between the two measures. However, implicit- and explicit-memory are supported by distinct neural pathways (e.g., Rugg et al., 1998; Schott et al., 2005, 2006). Thus, it is also plausible that value may *separately* enhance implicit- and explicit-memory, and such enhancements would be uncorrelated or negatively correlated (also see May et al., 2005; Gopie et al., 2011). Our results favored the latter hypothesis: implicit- and explicit-reward-based enhancements were negatively correlated across participants. In other words, participants who demonstrated greater reward value facilitation in lexical decision had *less* reward facilitation in free recall. Prior research also supports the notion of different value-based learning strategies leading to differential engagement of between implicit- and explicit-memory (Wimmer and Shohamy, 2011, see also Bayley et al., 2005). This apparent trade-off between memory systems due to learning strategy is also supported by research on the effects of stress on memory, where deliberative (i.e., goal-directed) and procedural (i.e., habit-based) learning strategies can similarly be learned through two distinct memory systems (Schwabe and Wolf, 2011; Schwabe et al., 2011a,b,c).

One possible source of this negative correlation may be behavior during the value-learning task, as suggested by the correlation analyses involving the trials-to-criterion measure. Trials-to-criterion explained the negative correlation between the effects of reward value on implicit- and explicit-memory. We suggest that the effects of value on implicit memory benefited from participants having more experience with the knowledge of the values of items. That is, the earlier someone learned to prefer the high- over the low-rewarding item during the training task, the more exposures to correct pairings of their own choice and high rewards they would have had. Such increased exposure and procedural practice of correct response-high reward pairings could then have selectively promoted the formation of an implicit memory bias. In contrast, free recall is a self-cued memory task; thus, value would be expected to influence free recall insofar as a participant includes value as part of their retrieval cue. Participants who initially found the value-learning task more challenging may have been oriented more toward value during the free-recall test, thus producing a positive relationship between trials-to-criterion and the effect of value free recall, opposite to what was observed with the effect of value on lexical decision. This indirect evidence of two distinct value-learning mechanisms may be related to similar dissociations in probabilistic value-learning strategies reported by others (Humphreys et al., 1968; Allen and Estes, 1972; Estes, 1972; Medin, 1972a).

Although lexical decision and free recall test implicit- and explicit-memory, respectively, the two tests also differ in several other ways, so alternative interpretations of the cause of the dissociation must be considered. First, the dependent measure in lexical decision was response time, a measure of access speed; in free recall, the dependent measure was probability of recall, a measure that is sensitive to sampling probability and recovery processes, as well as memory cueing processes (e.g., Raaijmakers and Shiffrin, 1981). Our dissociation could therefore reflect differential influences of reward value on access speed versus sampling, recovery or cueing processes. Second, participants are presented with a copy-cue to judge in lexical decision, but in free recall, participants must apply their own retrieval cues to generate responses. Our dissociation could thus reflect distinct influences of reward value on judgment processes versus item-retrieval processes (cf. Humphreys et al., 1989). Regardless of which of these accounts is correct, our findings extend the boundary conditions of reward-value enhancement of memory effects, and suggest that the effect of reward value on memory is non-unitary.

### 4.2 Influence of previously learned reward values on contextual binding

In Experiments 2a and 2b, what started as an advantage for high-value words (evident in Experiment 1) became a disadvantage when participants had to overcome proactive interference from the value-training phase and learn new sets of words that included both trained and untrained words. High- and low-value words were recalled at equivalent rates overall, but low-value words were produced earlier in output. High-value words were intruded more (and even more than expected based on the final free-recall rates of Experiment 1). These findings suggest less effective contextual binding for high- than for low-value words. This contradicts our reward-maximization hypothesis, and suggests that there are limits to the degree to which participants are biased to modulate their learning to maximize cumulative reward; one such limit is in relearning high-valued items in new, specific contexts.

If the additional resources devoted to high-value items included processing items within their context (i.e., the most recent list), then one would also expect participants to be able to rule out words that were recalled from previous contexts (i.e., the value-learning task or previous lists in the free-recall task), which is inconsistent with the elevated intrusion rate for high-value words in Experiments 2a and 2b. Thus, our findings are more consistent with our value-interference hypothesis, which posits that reward value impairs contextual binding. These results are also in line with findings obtained with manipulations of emotional arousal, where memory for the arousing items is enhanced, but the learning of new associations involving such items is impaired (Mather and Knight, 2008; Novak and Mather, 2009; Sakaki et al., 2011; Nashiro et al., 2012).

Although positive, as well as negative emotional items can be remembered better than emotionally neutral items (e.g., Dewhurst and Parry, 2000; Siddiqui and Unsworth, 2011), it would be reasonable to argue that the influence of reward value on memory may be more similar to the influence of positive – not negative – emotion on memory. While many studies have found that emotion can enhance memory for items and often impairs memory for associations, the majority of these findings used negatively valenced emotional stimuli (Fredrickson, 1998). Whereas negative emotions lead to attentional narrowing (e.g., the weapon focus effect; Loftus et al., 1987), positive emotion can to lead to a broadening of attention (Fredrickson, 1998). When participants are asked to learn associations containing emotionally positive, negative, or neutral items, participants are often better able to learn pairs with positive items than pairs with negative items (Zimmerman and Kelley, 2010; Okada et al., 2011; Pierce and Kensinger, 2011), suggesting that positive emotion can enhance participants’ ability to form associations between items. (Note that sometimes an association-memory impairment has been observed even with positive stimuli, e.g., Mather and Knight, 2008.) If this interpretation is correct, and reward value functions similar to positive emotionality, then one would expect reward value-based facilitation of free recall in Experiments 2a and 2b, inconsistent with our results. We recently showed previously reported arousal-based enhancements in association-memory could instead be attributed to enhanced memory for the target items, and that this item-memory effect can mask an underlying impairment of association-memory (Madan et al., 2012). Thus, it is similarly possible that prior findings regarding the effects of positive emotion on associative learning may be composed of conflicting effects. Finally, false memories can be viewed as failures of contextual discrimination. Emotion, both induced in the participant, and emotionality of items, can increase rates of false memories. This has been found for both negative and positive emotions (Storbeck and Clore, 2005; Corson and Verrier, 2007; Dehon et al., 2010), and appears similar to the list-discrimination problems we found for high-value items here.

### 4.3 Implications for previous findings of reward-value enhancements of memory

Reconsidering Raymond and O’Brien (2009) we detailed in the Introduction, our results suggest that their findings may have resulted from a summation of two distinct enhancement effects, one acting on implicit and the other acting on explicit memory. Regarding studies that have found that participants can prioritize their memory processes based on specific item-values presented alongside stimuli (Harley, 1965; Tarpy and Glucksberg, 1966; Weiner and Walker, 1966; Bjork and Woodward, 1973; Eysenck and Eysenck, 1982; Castel et al., 2002; Adcock et al., 2006; Gruber and Otten, 2010; Kuhl et al., 2010; Soderstrom and McCabe, 2011; Watkins and Bloom, unpublished manuscript), advantages in recall and recognition for high-value items resemble the enhancement effect we found in the final free-recall measure of Experiment 1. However, in all these studies, values were presented with items, but participants were never asked to link those items to a new context. Our findings in study/test free recall in Experiments 2a and 2b raise the possibility that if participants have to learn new lists composed of previously prioritized items, their memory might be compromised by the kind of value-based interference effect found here. In particular, given that the intrusion pattern was the largest effect we observed in Experiments 2a and 2b, we would predict that items previously linked to higher values would be intruded more – that is, participants would continue to produce them as responses even when inappropriate. In turn, since prioritization procedures directly ask participants to favor high-value items, whereas our procedure did not, it is quite possible that the list-discrimination procedure we found for high-value words could be overcome again if participants were asked to prioritize high-value words in later list learning.

## 5 Conclusion

Reward value can enhance memory for higher-valued items by increasing access speed and probability of retrieval. These dual enhancement effects of value on implicit- and explicit-memory measures may, in turn, be the results of dual value-learning styles. These enhancement effects come with a side effect of a poorer ability for participants to bind high-value items uniquely to a specific context, suggesting that items with high reward value can have a deleterious effect on subsequent memory tasks.

## Conflict of Interest Statement

The authors declare that the research was conducted in the absence of any commercial or financial relationships that could be construed as a potential conflict of interest.

## Footnotes

^1^Our criteria regarding the ANEW were to exclude words with an arousal rating greater than 5.5 scored on a scale from 1 (not arousing) to 9 (highly arousing), and a valence rating (also on a scale from 1 to 9) of either (a) less than 4 (negative), or (b) greater than 7 (positive). Note that we chose to keep two words that did meet the exclusion criteria: “dancer” and “rescue.”

## References

[B1] Abler B. Walter H. Erk S. (2005). Neural correlates of frustration. Neuroreport 16, 669–672 10.1097/00001756-200505120-00003 15858403

[B2] Adcock R. A. Thangavel A. Whitfield-Gabrieli S. Knutson B. Gabrieli J. D. (2006). Reward-motivated learning: mesolimbic activation precedes memory formation. Neuron 50, 507–517 10.1016/j.neuron.2006.03.036 16675403

[B3] Allen G. A. Estes K. (1972). Acquisition of correct choices and value judgements in binary choice learning with differential rewards. Psychon. Sci. 27, 68–72

[B4] Bayley P. J. Frascino J. C. Squire L. R. (2005). Robust habit learning in the absence of awareness and independent of the medial temporal lobe. Nature 436, 550–553 10.1038/nature03857 16049487 PMC1457096

[B5] Bijleveld E. Custers R. Aarts H. (2009). The unconscious eye opener: pupil dilation reveals strategic recruitment of resources upon presentation of subliminal reward cues. Psychol. Sci. 20, 1313–1315 10.1111/j.1467-9280.2009.02443.x 19788532

[B6] Bijleveld E. Custers R. Aarts H. (2010). Unconscious reward cues increase invested effort, but do not change speed-accuracy tradeoffs. Cognition 115, 330–335 10.1016/j.cognition.2009.12.012 20089247

[B7] Bjork R. A. Whitten W. B. (1974). Recency-sensitive retrieval processes in long-term free recall. Cogn. Psychol. 6, 173–189 10.1016/0010-0285(74)90009-7

[B8] Bjork R. A. Woodward A. E. Jr. (1973). Directed forgetting of individual words in free recall. J. Exp. Psychol. 99, 22–27 10.1037/h0034757

[B9] Bradley M. M. Lang P. J. (1999). Affective Norms for English Words (ANEW): Stimuli, Instruction Manual and Affective Ratings. Technical Report C-1. Gainesville, FL: The Center for Research in Psychophysiology, University of Florida

[B10] Brown G. D. A. Neath I. Chater N. (2007). A temporal ratio model of memory. Psychol. Rev. 114, 539–576 10.1037/0033-295X.114.2.528 17638496

[B11] Burke A. Heuer F. Reisberg D. (1992). Remembering emotional events. Mem. Cognit. 20, 277–290 10.3758/BF03199665 1508053

[B12] Castel A. D. Balota D. A. McCabe D. P. (2009). Memory efficiency and the strategic control of attention at encoding: Impairments of value-directed remembering in Alzheimer’s disease. Neuropsychology 23, 297–306 10.1037/a0014888 19413444 PMC2777518

[B13] Castel A. D. Benjamin A. S. Craik F. I. M. Watkins M. J. (2002). The effects of aging on selectivity and control in short-term recall. Mem. Cognit. 30, 1078–1085 10.3758/BF03194325 12507372

[B14] Castel A. D. Farb N. Craik F. I. M. (2007). Memory for general and specific value information in younger and older adults: measuring the limits of strategic control. Mem. Cognit. 35, 689–700 10.3758/BF03193307 17848027

[B15] Christianson S.-A. (1992). Emotional-stress and eyewitness memory: a critical-review. Psychol. Bull. 112, 284–309 10.1037/0033-2909.112.2.284 1454896

[B16] Corson Y. Verrier N. (2007). Emotions and false memories: valence or arousal? Psychol. Sci. 18, 208–211 10.1111/j.1467-9280.2007.01874.x 17444912

[B17] Crowder R. G. (1976). Principles of Learning and Memory. Hillsdale, NJ: Lawrence Erlbaum Associates

[B18] Custers R. Aarts H. (2010). The unconscious will: how the pursuit of goals operates outside conscious awareness. Science 329, 47–50 10.1126/science.1188595 20595607

[B19] Dehon H. Laroi F. Van der Linden M. (2010). Affective valence influences participant’s susceptibility to false memories and illusory recollection. Emotion 10, 627–639 10.1037/a0019595 21038946

[B20] Dewhurst S. A. Parry L. A. (2000). Emotionality, distinctiveness, and recollective experience. Eur. J. Cogn. Psychol. 12, 541–551 10.1080/095414400750050222

[B21] Easterbrook J. A. (1959). The effect of emotion on cue utilization and the organization of behavior. Psychol. Rev. 66, 183–201 10.1037/h0047707 13658305

[B22] Estes W. K. (1962). “Theoretical treatments of differential reward in multiple-choice learning and two-person interactions,” in Mathematical Methods in Small Group Processes, eds Criswell J. Solomon H. Suppes P. (Stanford, CA: Stanford University Press), 133–149

[B23] Estes W. K. (1966). Transfer of verbal discriminations based on differential reward magnitudes. J. Exp. Psychol. 72, 276–283 10.1037/h0023450 4961105

[B24] Estes W. K. (1972). Reinforcement in human behavior: reward and punishment influence human actions via informational and cybernetic processes. Am. Sci. 60, 723–729 5086051

[B25] Eysenck M. W. Eysenck M. C. (1982). Effects of incentive on cued recall. Q. J. Exp. Psychol. A 34, 489–498 10.1080/14640748208400832

[B26] Frank M. J. O’Reilly R. C. Curran T. (2006). When memory fails, intuition reigns: midazolam enhances implicit inference in humans. Psychol. Sci. 17, 700–707 10.1111/j.1467-9280.2006.01769.x 16913953

[B27] Frank M. J. Seeberger L. C. O’Reilly R. C. (2004). By carrot or by stick: cognitive reinforcement learning in Parkinsonism. Science 306, 1940–1943 10.1126/science.1102941 15528409

[B28] Fredrickson B. L. (1998). What good are positive emotions? Rev. Gen. Psychol. 2, 300–319 10.1037/1089-2680.2.3.300 21850154 PMC3156001

[B29] Gopie N. Craik F. I. M. Hasher L. (2011). A double dissociation of implicit and explicit memory in younger and older adults. Psychol. Sci. 22, 634–640 10.1177/0956797611403321 21421935

[B30] Gradin V. B. Kumar P. Waiter G. Ahearn T. Stickle C. Milders M. Reid I. Hall J. Steele J. D. (2011). Expected value and prediction error abnormalities in depression and schizophrenia. Brain 134, 1751–1764 10.1093/brain/awr059 21482548

[B31] Gruber M. J. Otten L. J. (2010). Voluntary control over prestimulus activity related to encoding. J. Neurosci. 30, 9793–9800 10.1523/JNEUROSCI.0915-10.2010 20660262 PMC2929460

[B32] Hadley C. B. MacKay D. G. (2006). Does emotion help or hinder immediate memory? Arousal versus priority-binding mechanisms. J. Exp. Psychol. Learn. Mem. Cogn. 32, 79–88 10.1037/0278-7393.32.1.79 16478342

[B33] Harley W. F. (1965). The effect of monetary incentive in paired associate learning using a differential method. Psychon. Sci. 2, 377–378

[B34] Howard M. W. Kahana M. J. (1999). Contextual variability and serial position effects in free recall. J. Exp. Psychol. Learn. Mem. Cogn. 25, 923–941 10.1037/0278-7393.25.4.923 10439501

[B35] Hubert L. J. Levin J. R. (1978). Evaluating priority effects in free recall. Br. J. Math. Stat. Psychol. 31, 11–18 10.1111/j.2044-8317.1978.tb00583.x

[B36] Humphreys M. S. Allen G. A. Estes W. K. (1968). Learning of two-choice, differential reward problems with informational constraints on payoff combinations. J. Math. Psychol. 5, 260–280 10.1016/0022-2496(68)90075-8

[B37] Humphreys M. S. Bain J. D. Pike R. (1989). Different ways to cue a coherent memory system: a theory for episodic, semantic, and procedural tasks. Psychol. Rev. 96, 208–233 10.1037/0033-295X.96.2.208

[B38] Johnsrude I. S. Owen A. M. White N. M. Zhao W. V. Bohbot V. (2000). Impaired preference conditioning after anterior temporal lobe resection in humans. J. Neurosci. 20, 2649–2656 10729345 10.1523/JNEUROSCI.20-07-02649.2000 PMC6772234

[B39] Johnsrude I. S. Owen A. M. Zhao W. V. White N. M. (1999). Conditioned preference in humans: a novel experimental approach. Learn. Motiv. 30, 250–264 10.1006/lmot.1999.1031

[B40] Kuhl B. A. Shah A. T. DuBrow S. Wagner A. D. (2010). Resistance to forgetting associated with hippocampus-mediated reactivation during new learning. Nat. Neurosci. 13, 501–508 10.1038/nn.2498 20190745 PMC2847013

[B41] Loftus E. F. Loftus G. R. Messo J. (1987). Some facts about ‘weapon focus’. Law Hum. Behav. 11, 55–62 10.1007/BF01044839

[B42] Loftus G. R. Masson M. E. J. (1994). Using confidence intervals in within-subject designs. Psychon. Bull. Rev. 1, 476–490 10.3758/BF03210951 24203555

[B43] Loftus G. R. Wickens T. D. (1970). Effect of incentive on storage and retrieval processes. J. Exp. Psychol. 85, 141–147 10.1037/h0029537

[B44] Madan C. R. Caplan J. B. Lau C. S. M. Fujiwara E. (2012). Emotional arousal does not enhance association-memory. J. Mem. Lang. 66, 695–716 10.1016/j.jml.2012.04.001

[B45] Madan C. R. Spetch M. L. (2012). Is the enhancement of memory due to reward driven by value or salience? Acta Psychol. (Amst.) 139, 343–349 10.1016/j.actpsy.2011.12.010 22266252

[B46] Mather M. Knight M. (2008). The emotional harbinger effect: poor context memory for cues that previously predicted something arousing. Emotion 8, 850–860 10.1037/a0014087 19102596 PMC2728072

[B47] Mather M. Sutherland M. R. (2011). Arousal-biased competition in perception and memory. Perspect. Psychol. Sci. 6, 114–133 10.1177/1745691611400234 21660127 PMC3110019

[B48] May C. P. Hasher L. Foong N. (2005). Implicit memory, age, and time of day: paradoxical priming effect. Psychol. Sci. 16, 96–100 10.1111/j.0956-7976.2005.00788.x 15686574 PMC1751473

[B49] Medin D. L. (1972a). Partial information and choice behaviour in differential reward magnitude learning. Psychon. Sci. 27, 73–76

[B50] Medin D. L. (1972b). Role of reinforcement in discrimination learning set in monkeys. Psychol. Bull. 77, 305–318 10.1037/h0032548

[B51] Nashiro K. Sakaki M. Huffman D. Mather M. (2012). Both younger and older adults have difficulty updating emotional memories. J. Gerontol. B. Psychol. Sci. Soc. Sci. 10.1093/geronb/gbs039 22451483 PMC3578257

[B52] Novak D. L. Mather M. (2009). The tenacious nature of memory binding for arousing negative items. Mem. Cognit. 37, 945–952 10.3758/MC.37.7.945 19744934 PMC2743275

[B53] Okada G. Okamoto Y. Kunisato Y. Aoyama S. Nishiyama Y. Yoshimura S. Onoda K. Toki S. Yamashita H. Yamawaki S. (2011). The effect of negative and positive emotionality on associative memory: an fMRI study. PLoS ONE 6, e24862 10.1371/journal.pone.0024862 21935483 PMC3173490

[B54] Pessiglione M. Schmidt L. Draganski B. Kalisch R. Lau H. Dolan R. J. Frith C. D. (2007). How the brain translates money into force: a neuroimaging study of subliminal motivation. Science 316, 904–906 10.1126/science.1140459 17431137 PMC2631941

[B55] Pessiglione M. Seymour B. Flandin G. Dolan R. J. Frith C. D. (2006). Dopamine-dependent prediction errors underpin reward-seeking behaviour in humans. Nature 442, 1042–1045 10.1038/nature05051 16929307 PMC2636869

[B56] Pierce B. H. Kensinger E. A. (2011). Effects of emotion on associative recognition: valence and retention interval matter. Emotion 11, 139–144 10.1037/a0021287 21401233 PMC3106271

[B57] Pubols B. H. (1960). Incentive magnitude, learning, and performance in animals. Psychol. Bull. 57, 89–115 10.1037/h0042065 14435211

[B58] Raaijmakers J. G. W. Shiffrin R. M. (1981). Search of associative memory. Psychol. Rev. 88, 93–134 10.1037/0033-295X.88.2.93

[B59] Raymond J. E. O’Brien J. L. (2009). Selective visual attention and motivation. Psychol. Sci. 20, 981–988 10.1111/j.1467-9280.2009.02391.x 19549080

[B60] Rugg M. D. Mark R. E. Walla P. Schloerscheidt A. M. Birch C. S. Allan K. (1998). Dissociation of the neural correlates of implicit and explicit memory. Nature 392, 595–598 10.1038/33396 9560154

[B61] Sakaki M. Niki K. Mather M. (2011). Updating existing emotional memories involves the frontopolar/orbito-frontal cortex in ways that acquiring new emotional memories does not. J. Cogn. Neurosci. 23, 3498–3514 10.1162/jocn_a_00057 21568639 PMC3203542

[B62] Schott B. H. Henson R. N. Richardson-Klavehn A. Becker C. Thoma V. Heinze H.-J. Düzel E. (2005). Redefining implicit and explicit memory: the functional neuroanatomy of priming, remembering, and control of retrieval. Proc. Natl. Acad. Sci. U.S.A. 102, 1257–1262 10.1073/pnas.0507123102 15657126 PMC545864

[B63] Schott B. H. Richardson-Klavehn A. Henson R. N. A. Becker C. Heinze H.-J. Düzel E. (2006). Neuroanatomical dissociation of encoding processes related to priming and explicit memory. J. Neurosci. 26, 792–800 10.1523/JNEUROSCI.3463-05.2006 16421299 PMC6675357

[B64] Schwabe L. Dickinson A. Wolf O. T. (2011a). Stress, habits, and drug addiction: a psychoneuroendocrinological perspective. Exp. Clin. Psychopharmacol. 19, 53–63 10.1037/a0022212 21341923

[B65] Schwabe L. Höffken O. Tegenthoff M. Wolf O. T. (2011b). Preventing the stress-induced shift from goal-directed to habit action with a β-adrenergic antagonist. J. Neurosci. 31, 17317–17325 10.1523/JNEUROSCI.3304-11.2011 22114298 PMC6623866

[B66] Schwabe L. Joëls M. Roozendaal B. Wolf O. T. Oitzl M. S. (2011c). Stress effects on memory: an update and integration. Neurosci. Biobehav. Rev. 36, 1740–1749 10.1016/j.neubiorev.2011.07.002 21771612

[B67] Schwabe L. Wolf O. T. (2011). Stress-induced modulation of instrumental behavior: from goal-directed to habitual control of action. Behav. Brain Res. 219, 321–328 10.1016/j.bbr.2010.12.038 21219935

[B68] Sescousse G. Redouté J. Dreher J.-C. (2010). The architecture of reward value coding in the human orbitofrontal cortex. J. Neurosci. 30, 13095–13104 10.1523/JNEUROSCI.3501-10.2010 20881127 PMC6633499

[B69] Shaoul C. Westbury C. (2006). USENET Orthographic Frequencies for the 40,481 Words in the English Lexicon Project. Edmonton, AB: University of Alberta

[B70] Shohamy D. Adcock R. A. (2010). Dopamine and adaptive memory. Trends Cogn. Sci. (Regul. Ed.) 14, 464–472 10.1016/j.tics.2010.08.002 20829095

[B71] Siddiqui A. P. Unsworth N. (2011). Investigating the role of emotion during the search process in free recall. Mem. Cognit. 39, 1387–1400 10.3758/s13421-011-0125-9 21713561

[B72] Soderstrom N. C. McCabe D. P. (2011). The interplay between value and relatedness as bases for metacognitive monitoring and control: evidence for agenda-based monitoring. J. Exp. Psychol. Learn. Mem. Cogn. 37, 1236–1242 10.1037/a0023548 21574750

[B73] Staudinger M. R. Erk S. Walter H. (2011). Dorsolateral prefrontal cortex modulates striatal reward encoding during reappraisal of reward anticipation. Cereb. Cortex 21, 2578–3588 10.1093/cercor/bhr041 21459835

[B74] Storbeck J. Clore G. L. (2005). With sadness comes accuracy; with happiness, false memory: mood and the false memory effect. Psychol. Sci. 16, 785–791 10.1111/j.1467-9280.2005.01615.x 16181441

[B75] Tarpy R. M. Glucksberg S. (1966). Effects of incentive and incentive – cue position on short-term retention. Psychon. Sci. 5, 313–314

[B76] Valentin V. V. O’Doherty J. P. (2009). Overlapping prediction errors in dorsal striatum during instrumental learning with juice and money reward in the human brain. J. Neurophysiol. 102, 3384–3391 10.1152/jn.91195.2008 19793875

[B77] Voon V. Pessiglione M. Brezing C. Gallea C. Fernandez H. H. Dolan R. J. Hallett M. (2010). Mechanisms underlying dopamine-mediated reward bias in compulsive behaviors. Neuron 65, 135–142 10.1016/j.neuron.2009.12.027 20152119 PMC2822730

[B78] Weber E. U. Johnson E. J. (2006). “Constructing preferences from memory,” in The Construction of Preference, eds Lichtenstein S. Slovic P. (New York: Cambridge, University Press), 397–410

[B79] Weiner B. (1966). Motivation and memory. Psychol. Monogr. Gen. Appl. 80, 1–22 10.1037/h0093909 5971462

[B80] Weiner B. Walker E. L. (1966). Motivational factors in short-term retention. J. Exp. Psychol. 71, 190–193 10.1037/h0022848 5903006

[B81] Westbury C. Hollis G. Shaoul C. (2007). LINGUA: the language-independent neighbourhood generator of the University of Alberta. Ment. Lex. 2, 273–286

[B82] Wilson M. D. (1988). The MRC psycholinguistic database: machine readable dictionary, version 2. Behav. Res. Methods 20, 6–11 10.3758/BF03202594

[B83] Wimmer G. E. Shohamy D. (2011). “The striatum and beyond: hippocampal contributions to decision making,” in Attention & Performance XXII, eds Delgado M. Phelps E. A. Robbins T. W. (Oxford: Oxford University Press), 281–310

[B84] Wittmann B. C. Dolan R. J. Düzel E. (2011). Behavioral specifications of reward-associated long-term memory enhancement in humans. Learn. Mem. 18, 296–300 10.1101/lm.1996811 21502336 PMC3465832

[B85] Wittmann B. C. Schott B. H. Guderian S. Frey J. U. Heinze H. J. Düzel E. (2005). Reward-related fMRI activation of dopaminergic midbrain is associated with enhanced hippocampus-dependent long-term memory formation. Neuron 45, 459–467 10.1016/j.neuron.2005.01.010 15694331

[B86] Wolosin S. M. Zeithamova D. Preston A. R. (2012). Reward modulation of hippocampal subfield activation during successful associative encoding and retrieval. J. Cogn. Neurosci. 24, 1532–1547 10.1162/jocn_a_00237 22524296 PMC3393089

[B87] Zimmerman C. A. Kelley C. M. (2010). “I’ll remember this!” effects of emotionality on memory predictions versus memory performance. J. Mem. Lang. 62, 240–253 10.1016/j.jml.2009.11.004

